# Polymethoxy-1-alkenes from *Aphanizomenon ovalisporum* Inhibit Vertebrate Development in the Zebrafish (*Danio rerio*) Embryo Model

**DOI:** 10.3390/md10102322

**Published:** 2012-10-22

**Authors:** Asha Jaja-Chimedza, Miroslav Gantar, Patrick D. L. Gibbs, Michael C. Schmale, John P. Berry

**Affiliations:** 1 Marine Science Program, Department of Chemistry and Biochemistry, 354 Marine Science Building, Florida International University, 3000 NE 151st Street, North Miami, FL 33181, USA; Email: ajaja001@fiu.edu; 2 Department of Biological Sciences, Florida International University, 11200 SW 8th Street, Miami, FL 33199, USA; Email: gantarm@fiu.edu; 3 Division of Marine Biology and Fisheries, Rosenstiel School of Marine and Atmospheric Science, University of Miami, 4600 Rickenbacker Causeway, Miami, FL 33149, USA; Email: pgibbs@rsmas.miami.edu (P.D.L.G.); mschmale@rsmas.miami.edu (M.C.S.)

**Keywords:** cyanobacteria, *Aphanizomenon ovalisporum*, toxins, zebrafish (*Danio rerio*) embryo, polymethoxy-1-alkenes, vertebrate development, harmful algal blooms (HABs)

## Abstract

Cyanobacteria are recognized producers of a wide array of toxic or otherwise bioactive secondary metabolites. The present study utilized the zebrafish (*Danio rerio*) embryo as an aquatic animal model of vertebrate development to identify, purify and characterize lipophilic inhibitors of development (*i.e.*, developmental toxins) from an isolate of the freshwater cyanobacterial species, *Aphanizomenon ovalisporum*.Bioassay-guided fractionation led to the purification, and subsequent chemical characterization, of an apparent homologous series of isotactic polymethoxy-1-alkenes (**1**–**6**), including three congeners (**4**–**6**) previously identified from the strain, and two variants previously identified from other species (**2** and **3**), as well as one apparently novel member of the series (**1**). Five of the PMAs in the series (**1**–**5**) were purified in sufficient quantity for comparative toxicological characterization, and toxicity in the zebrafish embryo model was found to generally correlate with relative chain length and/or methoxylation. Moreover, exposure of embryos to a combination of variants indicates an apparent synergistic interaction between the congeners. Although PMAs have been identified previously in cyanobacteria, this is the first report of their apparent toxicity. These results, along with the previously reported presence of the PMAs from several cyanobacterial species, suggest a possibly widespread distribution of the PMAs as toxic secondary metabolites and warrants further chemical and toxicological investigation.

## 1. Introduction

Cyanobacteria (“blue-green algae”) are known to produce a diverse repertoire of biologically active secondary metabolites [1]. When associated with so-called “harmful algal blooms”, particularly in freshwater systems, a number of these metabolites have been associated—as “toxins”, or commonly “cyanotoxins”—with human and animal health concerns [1,2,3]. The most widely recognized of the cyanobacterial toxins currently include hepatotoxic peptides (*i.e.*, microcystins, nodularins) alkaloids (*i.e.*, cylindrospermopsin), and neurotoxic alkaloids, including both representatives of the “saxitoxin family” of voltage-gated sodium channel inhibitors, and inhibitors of acetylcholine neurotransmitter pathways (*i.e.*, anatoxin-a and anatoxin-a(s)). More recently, the amino acid analog, β-methylamino-L-alanine (BMAA), has been additionally linked to neurodegenerative diseases [4], although this remains to some extent controversial [5]. As health concerns have largely been associated with exposure to drinking water contaminated with these toxins (except BMAA), it is notable, although perhaps not surprising, that all of these recognized toxins are generally polar and water-soluble metabolites.

In several previous studies [6,7,8], we have utilized the zebrafish (*Danio rerio*) embryo, as a model of vertebrate development, and specifically a toxicological (*i.e.*, bioassay) system, to investigate “developmental toxicity” (*i.e.*, inhibition or impairment of developmental pathways) of cyanobacterial secondary metabolites. The zebrafish embryo has become an increasingly important vertebrate model owing to a number of practical advantages including: a high fecundity capable of producing hundreds of eggs with a single breeding; rapid embryonic development with most internal organs being fully differentiated by 96 h post fertilization (hpf); as well as a nearly transparent outer chorion allowing for development to be easily monitored using a light microscope [7]. As such, this model system is finding utility in fields ranging from genetics to toxicology/pharmacology to biotechnology [9,10]; in our prior studies [6,7,8], the zebrafish embryo assay has been specifically applied to identification (*i.e.*, screening), purification (*i.e.*, bioassay-guided fractionation) and characterization of developmental toxins from cyanobacteria. 

As part of this on-going research, we previously evaluated [8] the cyanotoxin, cylindrospermopsin (CYN), as well as extracts from unialgal cultures of several CYN producing and non-producing strains of the widespread toxigenic cyanobacterial species, *Cylindrospermopsis raciborskii* and *Aphanizomenon ovalisporum*. Although CYN was found to be toxic, apparent impermeability of the embryonic chorion and/or cellular membranes, with respect to the water-soluble toxin, necessitated microinjection of the pure compound in order for toxicity to be observed. In fact, a similar apparent impermeability—and requirement for microinjection—was, likewise, observed for the equally water-soluble microcystins [11]. Moreover, toxicity of microinjected CYN was generally only associated with a dose-dependent increase in mortality of embryos, but no clear or reproducible developmental dysfunction [8]. In contrast, however, concurrent evaluation of crude, non-polar (*i.e.*, chloroform) extracts from strains of both *C. raciborskii* and *A. ovalisporum* identified developmental toxicity, clearly unrelated to the presence of CYN, and specifically associated with apparent lipophilic metabolites from extracts of all of the isolates evaluated [8].

**Figure 1:**
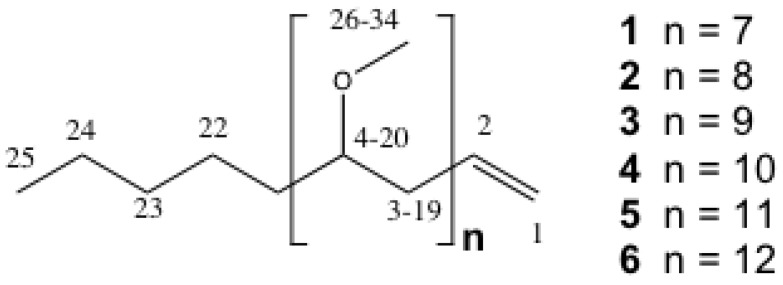
Structure of polymethoxy Alkenes (**1**–**6**) Isolated in the Current and Previous [13,14,15,16] Studies.

Toward understanding the possible contribution of these apparent lipophilic metabolites to the toxigenicity of these cyanobacterial species, the present study used the zebrafish embryo bioassay to guide purification for subsequent chemical and toxicological characterization of the previously identified [8] developmental toxin(s) from one of these isolates. Specifically, this study focused on a strain of *A. ovalisporum* previously isolated from Lake Kinneret, Israel, following a reported 1994 toxigenic bloom [12]. *Aphanizomenon*, as a genus, and particularly the species, *A. flos-aquae*, is a recognized producer of CYN, as well as the neurotoxic saxitoxin and anatoxin-a. Although *A. ovalisporum* is not known to produce either saxitoxin or anatoxin-a, studies of the Lake Kinneret isolate [12,13] identified both CYN and its 7-epimer (*i.e.*, 7-epi-CYN). Interestingly, and relevant to the present study, during the isolation of the latter CYN congener, a series of isotactic polymethoxylated*-*1*-*alkenes (**4**–**6**; Figure 1) were concurrently identified, purified and chemically characterized.

Indeed, in the present study, bioassay-guided fractionation, as described below, led to the isolation, and chemical characterization of these previously identified polymethoxy-1-alkenes (PMAs), *i.e.*, **4**–**6**, as well as three additional members (**1**–**3**) of the apparent homologous series (Figure 1), as lipophilic developmental toxins from the Lake Kinneret strain of *A. ovalisporum*. Although three of the six PMAs reported here have been isolated previously from this strain [13]—as well as at least two other genera (*i.e.*, *Scytonema*, *Tolypothrix*) of the cyanobacterial order Nostocales [14,15,16]—in studies spanning more than three decades, they have not previously been associated with toxicity. In addition to their isolation, and chemical characterization, we report here on their developmental toxicity in the zebrafish embryo model, and specifically, the apparent synergistic activity of congeners in the series.

## 2. Results and Discussion

### 2.1. Purification of PMAs by Bioassay-Guided Fractionation

**Figure 2:**
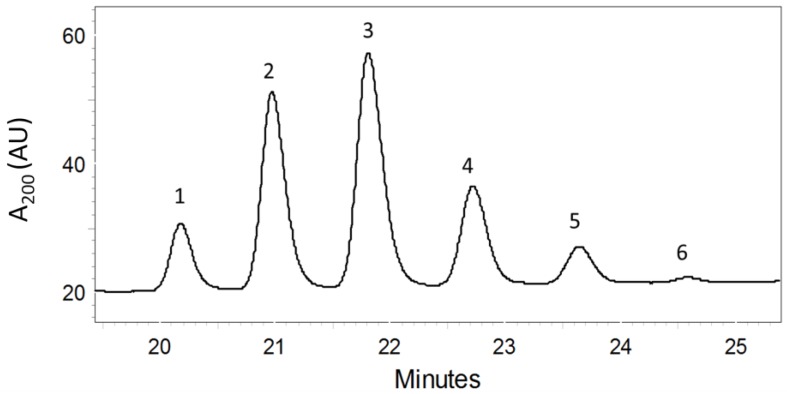
Chromatogram Showing PMAs (1–6) Isolated by HPLC. See Experimental Section for details.

Biomass from unialgal cultures of *A. ovalisporum* was (twice) extracted in CHCl_3_, and pooled extracts were confirmed to inhibit zebrafish embryo development as previously documented [8]. The pooled crude extracts were subsequently fractionated (see Experimental Section) by silica gel column chromatography and reverse-phase HPLC, affording a bioactive fraction containing a total of six components (**1**–**6**; Figure 2). LC-MS of the active fraction identified nominal masses of the presumptive molecular ions (M + H^+^) for compounds **1**–**6** of *m/z *505 (**1**), 563 (**2**), 621 (**3**), 679 (**4**), 737 (**5**) and 795 (**6**), in addition to apparent, *i.e.*, M + 23, sodium adducts (M + Na^+^) of each. An incremental mass difference of 58 amu, corresponding to -CH_2_CH(OCH_3_)- units, between each of the six compounds, as well as collision induced fragmentations, corresponding to sequential losses of 32 amu (-OCH_3_ lost as CH_3_OH) for each compound, was consistent with a homologous series of PMAs, as previously observed for the strain [13] as well as other cyanobacteria [14,15,16]. Each of the six peaks was collected for subsequent chemical and toxicological characterization (as described below).

### 2.2. Structure Elucidation of the PMAs

Compound **3** was the most abundant of the purified compounds and was isolated as a white amorphous solid. A molecular formula of C_34_H_68_O_9 _was suggested by high-resolution MS (HRMS; *m/z* 621.4920 [M + H]^+^), and subsequently confirmed by ^1^H and ^13^C-NMR studies (see below). MS/MS of the presumptive molecular ion (*m/z* 621.5) in the triple quadrupole instrument identified nine sequential losses of 32 amu consistent with nine methoxy groups (as MeOH) from the molecule. ^1^H NMR (Table 1), likewise, revealed eight signals at δ 3.1641 (s, 3H), 3.2105 (s, 3H), 3.218 (s, 3H), 3.2432 (s, 3H), 3.2514 (s, 3H), 3.2708 (s, 3H), 3.2745 (s, 3H) and 3.2798 (s, 6H), consistent with nine methoxy groups, with two of the methoxy groups being equivalent. Protons on the terminal alkene were identified at δ_H_ 5.09 (H-1*E*), 5.13 (H-1*Z*) and 5.93 (H-2), and three methyl protons tentatively assigned to C-25 (Figure 1) were observed at δ_H _0.91. Two sets of overlapping multiplets at δ 3.37 (m, 2H) and 3.65 (m, 7H) (Table 1) were assigned as oxymethine hydrogens geminal to the methoxy groups, as confirmed by subsequent HMBC experiments (see below). Likewise, overlapping multiplets were observed at δ 2.04 (m, 8H), 1.80 (m, 6H), 1.67 (m, 2H), 1.56 (m, 2H), 1.43 (m, 2H) and 1.30 (m, 4H), which integrated to a total of 24 protons. Based on distinct chemical shifts, and the assumption that **3** was a PMA, 16 of these were tentatively assigned to the protons vicinal to methoxy groups (δ 2.04, 1.80 and 1.67), and the remaining eight protons (δ 1.56, 1.43 and 1.30) were assigned to methylenes at C-21 to C-24 positions (Figure 1). Two additional methylene protons (δ 2.30) were identified and assigned to the C-3 position (Figure 1), specifically based on COSY correlation (see below) to the terminal alkene protons. Tentative proton assignments, and the proposed molecular formula, were confirmed by ^13^C-NMR and DEPT, as well as subsequent HMQC and HMBC experiments, as described below.

**Table 1:** ^1^H (C_6_D_6_, 400.13MHz) and ^13^C (C_6_D_6_, 100.61MHz) NMR Data for 4,6,8,10,12,14,16,18,20-nonamethoxy-1-pentacosene (**3**).

C#	δ_C_	δ_H_ (mult, *J* in Hz; #H)	COSY
1	117.01	5.13 (dd, 17, 2; 1H)	H-2
		5.09 (dd, 10, 2; 1H)	H-2
2	135.26	5.93 (ddt, 17, 10, 7; 1H)	H-1, H_2_-3
3	38.66	2.30 (m; 2H)	H-2, H-4
4	77.61	3.37 (m; 1H)	H-3, H_2_-5
5, 7, 9…19	38.35–38.97	1.67–2.04 (m; 16H)	See text
6, 8, 10…18	75.92–76.11	3.65 (m; 7H)	See text
20	78.13	3.37 (m; 1H)	H_2_-21, H_2_-19
21	33.96	1.56 (m; 2H)	H_2_-20, H_2_-22
22	25.09	1.43 (m; 2H)	H_2_-21, H_2_-23
23	32.51	1.30 (m; 2H)	H_2_-22, H_2_-24
24	23.08	1.30 (m; 2H)	H_2_-23, H_2_-25
25	14.27	0.91 (t, 7.1; 3H)	H_2_-24
26–34	55.93–56.16	See text	

COSY experiments, in conjunction with HMQC and HMBC, revealed the apparent linear structure of **3**. Protons of the single methyl group (H_3_-25, δ 0.91) were found to correlate by COSY to two pairs of hydrogens (δ 1.30) that were subsequently revealed, by HMQC correlations, to be bonded to two methylene carbons. HMBC correlation of both methylene carbons to protons of the methyl group further enabled assignment of C-25, -24 and -23 (as shown in Figure 1). COSY correlation of C-23/24 protons to a single pair of methylene protons (assigned as C-22), and subsequent correlation to a single adjacent pair of methylene protons (assigned as C-21)—as well as COSY correlation of C-21 hydrogens to a pair of oxymethine protons (δ 3.37)—enabled assignments of hydrogens on C-25 through C-20 as shown in Figure 1. Similarly, at the alkene terminal (Figure 1), COSY correlation was observed between alkene hydrogens and a single pair of methylene protons that was consequently assigned to C-3. Although methylene protons on both C-21 and C-3 correlated to the same two overlapping oxymethine proton signals (assigned C-20 and C-4, respectively), correlation of these protons to C-22 methylene and C-1/2 terminal alkene protons, respectively, allowed these two pairs of methylene hydrogens to be clearly distinguished. 

Finally, a stretch of nine alternating methoxy-bearing carbons and the methylene carbons between (C-4 to C-20; Figure 1) were identified based on correlations in COSY and HMBC. HMBC correlations between proposed methoxy carbons (nine in total) and seven remaining oxymethine protons, along with mass fragmentation (*i.e.*, loss of 32 amu) consistent with –OCH_3 _functional groups, further confirmed their identity as methoxy-bearing carbons. Chemical shifts, in both ^1^H- and ^13^C-NMR spectra, assigned to protons and carbons of the remaining methylenes and oxymethines, overlapped considerably, and did not consequently allow resolution of these signals for direct correlations. However, COSY and HMBC correlation of the total integrated signals for the methylene and oxymethine protons (*i.e.*, 16 and 9, respectively) and corresponding carbons (*i.e.*, 8 and 9, respectively) confirmed that methoxy-bearing carbons alternated with methylenes. 

Accordingly, **3 **was determined to be 4,6,8,10,12,14,16,18,20-nonamethoxy-1-pentacosene. As such, this compound would represent a structural isomer of 4,6,8,10,12,14,16,18,22-nonamethoxy-1-pentacosene previously isolated from the same strain by Banker *et al.* [13]. Although a new PMA variant for this particular strain, **3** was, in fact, previously isolated by Mynderse and Moore [14] from *Tolypothrix conglutinata *var. *colorata*—specifically as the similarly most abundant (~80%) of three congeners isolated from this strain—as well as by Mori *et al.* [15] from *Scytonema burmanicum*. In this case, as in other prior studies [13,15,16], methoxyl groups of the PMA were determined, based on NMR studies, to be isotactic (*i.e.*, on the same side of the alkene chain). Based on the nearly identical ^1^H- and ^13^C-NMR observed here, **3** was proposed to be, likewise, isotactic.

Although quantities of each of the less abundant PMAs purified, including the more major (*i.e.*, **2** and **4**) and minor (*i.e.*, **1**, **5** and **6**; see Figure 2) congeners, did not generally allow for ^13^C-NMR and related NMR analysis (*i.e.*, HMQC, HMBC), structures were assigned based on comparison to the ^1^H-NMR and associated assignments based on COSY of **3 **(as described above), as well as MS/MS (*i.e.*, losses of 32 amu corresponding each methoxy group). Furthermore, comparison of this data (*i.e.*, ^1^H-NMR, COSY, MS) to that previously published and available for characterization of PMAs, and specifically, studies by Mynderse and Moore [14] and Mori *et al.* [15,16], were used to support these structural assignments. A clear correlation (e.g., nearly identical ^1^H-NMR chemical shifts, identical molecular ions and subsequent fragmentation patterns) between spectroscopic data obtained for **2** and **4**–**6** in the present study, as well as those previously reported [13,14,15,16], support our structural determinations for these less abundant variants. On the other hand, although **1** has not been previously identified from any other source, consistency in the NMR and MS data for this congener, compared to the other purified in the present study, strongly support the proposed structure of this variant. Similar to **3**, the stereochemistry—and specifically the assignment as isotactic methoxy groups—was, likewise, concluded based on the similarities observed in the NMR data for these congeners and those previously characterized [13,15,16]. Accordingly, **1 ** and **2** were identified as isotactic 4,6,8,10,12,14,16-heptamethoxy-1-uncosene and 4,6,8,10,12,14,16,18-octamethoxy-1-tricosene, respectively. The former variant (**1**) has not been previously isolated (to the authors’ knowledge) from cyanobacteria, however, the latter (**2**) was previously isolated from *T. conglutinata * [14]. On the other hand, **4**–**6** were identified as 4,6,8,10,12,14,16,18,20,22-decamethoxy-1-heptacosene, 4,6,8,10,12,14,16,18,20,22,24-undecamethoxy-1-nonacosene and 4,6,8,10,12,14,16,18,20,22,24,26-dodecamethoxy-1-hentriacontene, respectively, and each has been previously isolated from the Lake Kinneret isolate of *A. ovalisporum *[13] and other cyanobacteria [15,16]. Additional members of the apparent homologous series, including penta- and hexa-methoxylated variants, although not seen here, have been previously isolated from species of *Scytonema* [15,16].

Interestingly, the linear polymethoxylated structure of the PMAs suggests a possible biosynthetic origin based on the polyketide synthase (PKS) pathway found conspicuously throughout the secondary metabolism of fungi, bacteria and microalgae [17]. Indeed, this biosynthetic route—and specifically the sequential condensation of acetate—for the PMAs has been previously suggested by other authors. Banker *et al.* [13], for example, proposed such a biosynthetic origin based on both consideration of structure, and the observation that PMAs were not found among *A. ovalisporum *cultures that lost their ability to produce the PKS-derived CYN. Similarly, both Mynderse and Moore [14] and Mori *et al.* [15,16] identified PMAs from strains of *Tolypothrix *and *Scytonema*, respectively, known to produce the polyketide, tolytoxin. Given the abundance of PKS-derived secondary metabolites among cyanobacteria and other microalgae, this biosynthetic origin may further support a widespread distribution of the PMAs.

### 2.3. Toxicological Studies of PMAs

Although previously isolated from *A. ovalisporum* [13], and several other cyanobacterial species [14,15,16], PMAs have not been previously associated with toxicity or other bioactivity. Indeed, Mynderse and Moore [14] first isolated the isotactic PMAs, including **2**, **3** and **4**, as components of “non-toxic mixture” while looking for the cytotoxic polyketide, tolytoxin A. As such, the present study is the first report of the biological activity of the PMAs. It should, however, be noted that a parallel series of polymethoxydienes isolated from the marine sponge, *Myriastra clavosa*, were found to be moderately cytotoxic to a human colon cancer cell-line, and suggested in fact, based on chemical similarity, to be derived (via diet and metabolic transformation) from cyanobacteria [18].

In the current study, five of the six PMAs (**1**–**5**) were obtained in sufficient (*i.e.*, accurately weighable) quantities, and subsequently evaluated for developmental toxicity in the zebrafish embryo model. Zebrafish embryos, exposed (five embryos per concentration) to a representative concentration range (10, 25, 50 and 100 µg/mL) of **1**–**5**, were observed up to five days post-fertilization (dpf) for mortality and apparent developmental dysfunction. It should be noted that solubility, and consequently the absolute dissolved concentration, of PMAs in aqueous assay medium remains uncertain; however, the assumption of relatively low water-solubility of these lipophilic compounds would suggest that prepared exposure concentrations may, in fact, overestimate actual dissolved exposure concentrations. 

Notably, toxicity of PMAs in the zebrafish embryo assay seemed to correlate with chain length and/or associated degree of methoxylation of the variants. With regards to embryo mortality (Table 2), no effect was observed at the lowest exposure concentrations (10 and 25 µg mL^−1^) for any of the variants tested. In a typical experiment, however, 100% mortality at 5 dpf was observed for embryos exposed to both 50 and 100 µg mL^−1^ of the three smallest congeners (**1**–**3**), whereas relatively little (*i.e.*, 20%–40%) or no mortality for the same doses were observed for embryos exposed to the two increasingly larger congeners (*i.e.*, **4** and **5**, respectively) tested. For comparison, mortality in untreated controls was very low (*i.e.*, approximately 1.7%, *n* = 60 embryos). Similarly, there was an apparently parallel, congener-dependent effect on hatching rates (Table 2). Again, little or no effect on hatching (*i.e.*, 80%–100% hatching rate at 4 dpf) was observed for lowest exposure concentrations (10 and 25 µg mL^−1^) of any of the PMAs tested relative to the untreated controls (98% hatching, *n* = 60 eggs). However, the hatching of embryos was inhibited by PMAs in a dose- and congener-dependent manner at the two highest exposure concentrations (50 and 100 µg mL^−1^). Specifically, hatching (4 dpf) was completely inhibited in embryos exposed to 100 µg mL^−1^ of congeners **1**–**3**, but only partially inhibited for embryos exposed to **4,** and not appreciably affected in embryos exposed to the largest variant (**5**). Supporting a dose and congener dependent effect, at 50 µg mL^−1^ hatching was completely inhibited by only the two smallest congeners (**1 **and **2**), and decreasingly affected by the larger **3**–**5.** These observations were further supported by a preliminary evaluation of **3**–**5,** which were initially purified in sufficient quantities to test. This preliminary exposure study showed a similar pattern to that reported in Table 2 for these congeners, with **3** causing 100% mortality as well as gross development defects at the highest concentrations tested (50 and 100 µg mL^−1^), while **4** caused only low mortality (20%) at the highest concentration (100 µg mL^−1^) and none at lower concentrations, and **5** did not cause mortality at any exposure level at 5 dpf. Although little or no mortality was observed for embryos exposed to **4** at these highest concentrations, all surviving embryos were characterized by developmental dysfunction and deformity (as discussed further below); in contrast, no distinguishable effects on development were observed for embryos exposed to any concentration of **5**. Thus, while the limited availability of purified material precluded conducting a full-scale quantitative toxicological characterization of the PMAs, the consistency of the trends observed strongly supports a dose and congener related toxicity effect on these embryos.

**Table 2:** Mortality and Hatching Rates for Zebrafish Embryos Exposed to PMAs (**1**–**5**) from *A. ovalisporum* (Lake Kinneret, Israel). Mortality was recorded at five days post-fertilization (dpf) for five embryos per concentration. Hatching rates were recorded at 4 dpf.

	% Mortality (5 dpf)^ b^				Hatching Rate (4 dpf)^ b^			
**PMA**								
**^a^** ** Conc.:**	**10**	**25**	**50**	**100**	**10**	**25**	**50**	**100**
**1**	20%	0%	**100%**	**100%**	100%	100%	**0%**	**0%**
**2**	0%	0%	**100%**	**100%**	100%	80%	**0%**	**0%**
**3**	0%	0%	**100%**	**100%**	100%	80%	**40%**	**0%**
**4**	0%	0%	**40%**	**20%**	100%	100%	**60%**	**60%**
**5**	0%	0%	**0%**	**0%**	100%	100%	**80%**	**80%**

^a^ Concentration of PMAs tested at 10, 25, 50 and 100 µg mL^−1^; ^b^ In untreated controls, mortality was approximately 1.7%, while hatching rate was 98% (*n* = 60 embryos).

**Figure 3:**
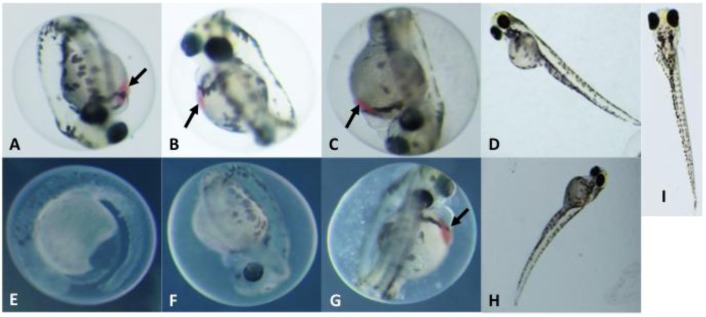
Developmental Toxicity of PMAs (1–4) Isolated from *A. ovalisporum* (Lake Kinneret, Israel). Photomicrographs of embryos at 4 dpf. Shown are embryos exposed to **1**–**4** at 50 µg mL^−1^ (**A**–**D**, respectively) and 100 µg mL^−1^ (**E**–**H**, respectively). Untreated control embryo at 4 dpf (**I**) shown for comparison. Arrows indicate areas where blood is pooled adjacent to the heart. Images in A–C and E–G, are not to scale with D, H and I.

**Figure 4:**
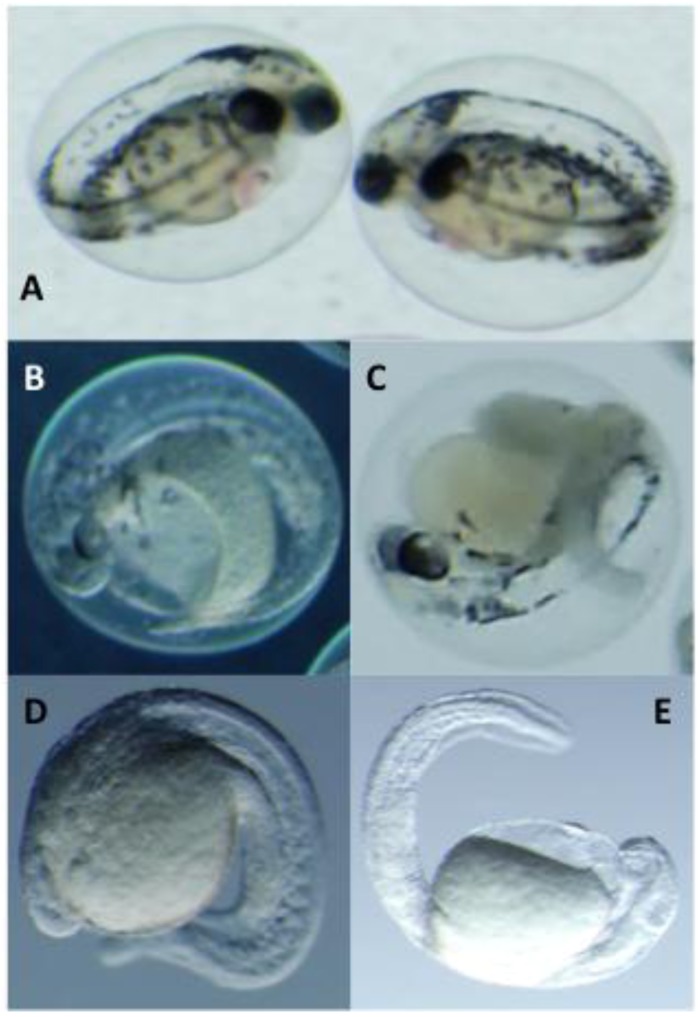
Apparent Synergistic Interactions in the Developmental Toxicity of 3 and 4. Shown are embryos exposed to 50 µg mL^−1^ of **3** (**A**), and 100 µg mL^−1^ of **3** (**B**) and **4** (**C**), compared to an equivalent combined total concentration (*i.e.*, 25 and 50 µg mL^−1^ of each) of the two variants (**D** and **E**, respectively). Embryos shown at 4 dpf.

In addition to the effects on mortality and hatching rate, there was also an apparent correlation between both exposure dose and structural variation of the PMAs (*i.e.*, degree of methoxylation, chain length) and observed morphological developmental effects (Figure 3). At the highest concentration tested (100 µg mL^−1^), embryos exposed to the smallest congener (**1**) exhibited several indications of severely impaired development (Figure 3E), including near lack of pigmentation, impaired organogenesis (e.g., reduced eye formation), deformity of the yolk sac and other gross abnormalities. Although developmental effects were less profound for congeners **2** and **3** at the same exposure concentration (100 µg mL^−1^), embryos exposed to both of these larger variants were also characterized (Figure 3F,G, respectively) by similar defects as well as the formation of edemas (along with reduced hatching rates; see above). Developmental dysfunction was uniformly observed (*i.e.*, 100% of embryos), although the severity of this dysfunction varied, and in each case, was followed by embryo mortality by 5–6 dpf. In contrast, embryos exposed to the highest dosage of congener **4** showed relatively limited developmental effects (e.g., slightly curved body axes; Figure 3H) and those exposed to **5** were indistinguishable (data not shown) from untreated controls (Figure 3I). Development was also inhibited and abnormal in all embryos exposed to a lower concentration (50 µg mL^−1^) of **1**–**3**, but there was no clear correlation of prevalence or severity of defects with congener size. (Figure 3A–C). However, similar to the effects observed at the higher concentrations, there were clearly minimal effects of congener **4** (Figure 3D) and no detectable effects associated with **5**. One particularly notable effect observed was an apparent pooling of blood between the anterior surface of the yolk and the heart in embryos exposed to **1**–**3** (indicated by arrows in Figure 3); this was most obvious in embryos exposed to the lower concentration (50 µg mL^−1^) of all three of these congeners and the higher concentration (100 µg mL^−1^) of the largest of these variants (**3**). This effect was not observed in the more severely deformed embryos exposed to the highest concentration of **1 **and **2**, where it was likely obscured by the numerous severe developmental defects present in these fish. As for the assessment of mortality and hatching rate (see above; Table 2), data from a single experiment are presented, due to limitations of purified PMAs. However, additional studies with a subset of these congeners, including both evaluation of initially purified amounts of **3**–**5 **(as discussed above), and the subsequent evaluation of **3** and **4** with respect to apparent interactive effects (Figure 4; discussed below), likewise generally confirm the observed congener-dependent trend of delayed and/or dysfunctional development.

Based on the qualitatively more pronounced developmental toxicity observed during bioassay-guided fractionation compared to that of individual congeners, we evaluated pair-wise combinations of **3** and **4**—as two of the most abundant variants (*i.e.*, purified in the largest quantity) with the latter showing only relatively limited toxicity—in order to assess possible synergistic and/or additive effects of the PMAs. Interestingly, a clear interactive effect of the congeners, in terms of embryos toxicity, was observed for embryos exposed to a combination of two variants when compared to either **3 **or **4**,individually. Embryos exposed to 50 and 100 µg mL^−1^ of each compound were characterized by the typically moderate developmental dysfunction observed for these variants (Figure 3 and Figure 4A–C). However, when exposed to equivalent total concentrations comprised of equal amounts congeners 3 and 4 (*i.e.*, 25 µg mL^−1^ and 50 µg mL^−1^, respectively, of **3** and **4**), there was a clearly more pronounced inhibition of development (Figure 4D,E). All embryos exposed to both combined concentrations were characterized by a severe inhibition of organogenesis and overall differentiation and development of the embryo that was not observed for embryos exposed to any of the congeners individually at an equivalent concentration. This observation is consistent with a synergistic effect of these variants and may explain increased toxicity of PMA mixtures (when compared to individual variants). Synergistic interactions of biologically active cyanobacterial metabolites have been previously reported. Recently, for example, Leão *et al.* [19] documented similar synergistic effects with regards to algicidal activity of the cyclic peptide congeners, portoamides A and B, isolated as apparent allelochemicals from a freshwater strain of *Oscillatoria*. Likewise, a similarly synergistic effect of lobocyclamides A and B, isolated from a marine cyanobacterial species, *Lyngbya confervoides*, was previously reported [20], specifically in relation to antifungal activity of these lipopeptides.

The identification of toxicity associated with the PMAs, combined with the previous isolation of related variants from other distantly related marine, freshwater and terrestrial cyanobacterial genera [14,15,16], as well as the cyanobacterially derived polymethoxydienes found in marine sponges [18], suggest that PMAs may represent a relatively widespread toxic metabolite from the blue-green algae. Moreover, preliminary evaluation by LC-MS, and subsequent purification and identification (by MS and ^1^H-NMR), confirmed the presence of **1**–**6** in the extracellular medium of the *A. ovalisporum* cultures (data not shown), and suggests that these lipophilic metabolites may, to some degree, be released—either actively by excretion, or passively via senescence of algal cells—into the surrounding aqueous environment. Accordingly, it is proposed that the presence of these lipophilic metabolites may not only contribute to previously observed toxicity associated with lipophilic fractions of toxigenic cyanobacteria [8], but to the observed toxicity of the bloom-forming cyanobacteria more generally.

## 3. Experimental Section

### 3.1. Culturing of the Cyanobacteria

The isolate of *A. ovalisporum*, (designated APH 127-1) was grown, as previously described [7,8,21], in 3-L flasks in BG11 medium under cool-white fluorescent light (intensity 30 µEm^−2^∙s^−1^) with aeration. The cells/biomass were harvested after three to four weeks by centrifugation and freeze-dried.

### 3.2. Extraction and Isolation of Polymethoxy-1-alkenes

Isolation and purification of the polymethoxy-1-alkenes was carried out by a bioassay-guided fractionation using the zebrafish embryo model. Freeze-dried biomass of *A. ovalisporum *was extracted in chloroform in a 10:1 ratio overnight. The filtered extract was initially fractionated by flash chromatography on a normal phase silica-gel glass column (Silica Gel 60Å, Commercial 40–63 μm) with a stepwise gradient of ethyl acetate in hexane; a bioactive fraction (as identified with the zebrafish embryo toxicity assay; see below) eluted with 100% ethyl acetate. This bioactive (*i.e.*, 100% ethyl acetate) fraction was further separated by reverse-phase HPLC (Phenomenex Luna 5 µm C18 100 Å LC Column 30 × 4.6 mm; solvent gradient of 50:50 acetonitrile/water to 100% acetonitrile for 37 min) with UV detection (*i.e.*, 200 nm absorbance). The crude mixture of all six polymethoxy-1-alkenes eluted with retention times between 21 and 24 min. This mixture of polymethoxy-1-alkenes was confirmed to be toxic in the zebrafish embryo model (see below), and individual PMAs were purified by HPLC-UV (isocratic 65:35 acetonitrile/water) with the compounds eluting in order of increasing size (see Figure 2). 

### 3.3. Zebrafish Embryo Bioassay

The zebrafish (*Danio rerio*) embryo was used as a model of developmental toxicity for both bioassay-guided fractionation and subsequent toxicological characterization. The maintenance and breeding of adult zebrafish, and the embryo toxicity bioassays, were carried out as previously described by Berry *et al*. [7,8] and according to protocols approved by the FIU Institutional Animal Care and Use Committee (IACUC). Toxicity was evaluated in solvent-resistant, polypropylene 24-well plates (Evergreen Scientific, Los Angeles, CA) with five embryos per well in E3 medium [22]. Extracts and fractions were typically evaluated at a high- (100 µL) and low- (10 µL) exposure concentration; purified PMAs were evaluated at concentrations of two, five, 10, 25, 50 and 100 µg/mL concentrations (in acetonitrile), and purified **3** and **4** were specifically evaluated in combination at 10, 25 and 50 µg/mL of each (20, 50 and 100 µg/mL total) to evaluate synergistic activity. Vehicle (*i.e.*, acetonitrile-only) and “untreated” controls, respectively, were included in each assay; the solvent was evaporated from assay plates, prior to adding medium and embryos, and accordingly there was no observed effect of the solvent (relative to the untreated controls). The embryos were observed up to 5 days post fertilization (dpf) for bioactivity. In addition to percent hatching and mortality, developmental toxicity was recorded by photomicrography using an Olympus SZX7 stereomicroscope equipped with an Olympus DP72 digital camera. 

### 3.4. Characterization of Polymethoxy-1-alkenes

Purified compounds were characterized by NMR and mass spectrometry. ^1^H- and ^13^C/DEPT-NMR, as well as two-dimensional homonuclear (*i.e.*, COSY) and heteronuclear (*i.e.*, HMQC and HMBC) analyses, were done on a Bruker AVANCE 400 MHz instrument. Mass spectrometry was performed on a Thermo TSQ Quantum Access ESI/triple quadrupole instrument coupled to a Thermo Accela UHPLC; high-resolution mass spectrometry data was done at the Scripps Florida Mass Spectrometry and Proteomics laboratory (Jupiter, FL) on a Thermo LTQ ESI-Orbitrap. 

**4,6,8,10,12,14,16-heptamethoxy-1-uncosene (1):** LCMS *m/z* 505 (C_28_H_56_O_7_, [M + H]^+^); ^1^H NMR (400 MHz, benzene-*d*_6_) δ 0.91 (t, 3H, *J* = 7.1 Hz), 1.30 (m, 4H), 1.43 (m, 2H), 1.56 (m, 2H), 1.67 (m, 2H), 1.81 (m, 4H), 2.02 (m, 6H), 2.30 (m, 2H), 3.16 (s, 3H), 3.21 (s, 3H), 3.22 (s, 3H), 3.24 (s, 3H), 3.25 (s, 3H), 3.27 (s, 3H), 3.27 (s, 3H), 3.37 (m, 2H), 3.64 (m, 5H), 5.07 (dd, 1H, *J* = 10,2 Hz), 5.09 (dd, 1H, *J* = 17, 2 Hz), 5.90 (ddt, 1H, *J* = 17, 10, 7 Hz).

**4,6,8,10,12,14,16,18-octamethoxy-1-tricosene (2):** LCMS *m/z* 563 (C_31_H_62_O_8_, [M + H]^+^); ^1^H NMR (400 MHz, benzene-*d*_6_) δ0.91 (t, 3H, *J* = 7.1 Hz), 1.30 (m, 4H), 1.42 (m, 2H), 1.56 (m, 2H), 1.67 (m, 2H), 1.81 (m, 5H), 2.03 (m, 7H), 2.30 (m, 2H), 3.16 (s, 3H), 3.21 (s, 3H), 3.22 (s, 3H), 3.24 (s, 3H), 3.25 (s, 3H), 3.27 (s, 3H), 3.27 (s, 3H), 3.28 (s, 3H), 3.37 (m, 2H), 3.64 (m, 6H), 5.07 (dd, 1H, *J* = 10, 2 Hz), 5.09 (dd, 1H, *J* = 17, 2 Hz), 5.90 (ddt, 1H, *J* = 17, 10, 7 Hz).

**4,6,8,10,12,14,16,18,20-Nonamethoxy-1-pentacosene (3):** LCMS *m/z* 621.4944 (C_34_H_68_O_9_, [M + H]^+^); ^1^H NMR (400 MHz, benzene-*d*_6_) δ 0.91 (t, 3H, *J* = 7.1 Hz), 1.30 (m, 4H), 1.43 (m, 2H), 1.56 (m, 2H), 1.67 (m, 2H), 1.80 (m, 6H), 2.04 (m, 8H), 2.30 (m, 2H), 3.1641 (s, 3H), 3.2105 (s, 3H), 3.218 (s, 3H), 3.2432 (s, 3H), 3.2514 (s, 3H), 3.2708 (s, 3H), 3.2745 (s, 3H), and 3.2798 (s, 6H), 3.37 (m, 2H), 3.65 (m, 7H), 5.09 (dd, 1H, *J* = 10, 2 Hz), 5.13 (dd, 1H, *J* = 17, 2 Hz), 5.93 (ddt, 1H, *J* = 17, 10, 7 Hz); ^13^C NMR (100 MHz, benzene-*d*_6_) δ 14.3, 23.1, 25.1, 32.5, 33.9, 38.0, 38.2, 38.3, 38.6, 38.6 (3C), 38.7 (4C), 55.9, 56.0, 56.0 (3C), 56.0 (2C), 56.1, 56.1, 75.6, 75.7 (2C), 75.7, 75.8, 77.6, 78.1, 117.0, 135.3.

**4,6,8,10,12,14,16,18,20,22-Decamethoxy-1-heptacosene (4):** LCMS *m/z* 679.5347 (C_37_H_74_O_10_, [M + H]^+^); ^1^H NMR (400 MHz, benzene-*d*_6_) δ 0.91 (t, 3H, *J* = 7.1 Hz), 1.30 (m, 4H), 1.44 (m, 2H), 1.58 (m, 2H), 1.68 (m, 2H), 1.81 (m, 7H), 2.03 (m, 9H), 2.30 (m, 2H), 3.16 (s, 3H), 3.21 (s, 3H), 3.22 (s, 3H), 3.24 (s, 3H), 3.25 (s, 3H), 3.27 (s, 3H), 3.27 (s, 3H), 3.28 (s, 9H), 3.37 (m, 2H), 3.64 (m, 8H), 5.07 (dd, 1H, *J* = 10, 2 Hz), 5.10 (dd, 1H, *J* = 17, 2 Hz), 5.90 (ddt, 1H, *J* = 17, 10, 7 Hz).

**4,6,8,10,12,14,16,18,20,22,24-Undecamethoxy-1-nonacosene (5):** LCMS *m/z* 737 (C_40_H_80_O_11_, [M + H]^+^); ^1^H NMR (400 MHz, benzene-*d*_6_) δ 0.91 (t, 3H, *J* = 7.1 Hz), 1.30 (m, 4H), 1.44 (m, 2H), 1.57 (m, 2H), 1.68 (m, 2H), 1.81 (m, 8H), 2.03 (m, 10H), 2.30 (m, 2H), 3.16 (s, 3H), 3.21 (s, 3H), 3.22 (s, 3H), 3.24 (s, 3H), 3.25 (s, 3H), 3.27 (s, 6H), 3.27 (s, 3H), 3.28 (s, 9H), 3.37 (m, 2H), 3.64 (m, 9H), 5.07 (dd, 1H, *J* = 10, 2 Hz), 5.10 (dd, 1H, *J* = 17, 2 Hz), 5.90 (ddt, 1H, *J* = 17, 10, 7 Hz).

**4,6,8,10,12,14,16,18,20,22,24,26-dodecamethoxy-1-hentriacontene (6):** LCMS *m/z* 795 (C_43_H_86_O_12_, [M + H]^+^); ^1^H NMR (400 MHz, benzene-*d*_6_) δ 0.91 (t, 3H, *J* = 7.1 Hz), 1.30 (m, 4H), 1.44 (m, 2H), 1.57 (m, 2H), 1.68 (m, 2H), 1.81 (m, 9H), 2.03 (m, 11H), 2.30 (m, 2H), 3.16 (s, 3H), 3.21 (s, 3H), 3.22 (s, 3H), 3.24 (s, 3H), 3.25 (s, 3H), 3.27 (s, 6H), 3.27 (s, 3H), 3.28 (s, 12H), 3.37 (m, 2H), 3.64 (m, 10H), 5.07 (dd, 1H, *J* = 10, 2 Hz), 5.10 (dd, 1H, *J* = 17, 2 Hz), 5.90 (ddt, 1H, *J* = 17, 10, 7 Hz).

## 4. Conclusions

In the present study, we used the zebrafish embryo model to identify, isolate (via bioassay-guided fractionation) and characterize a homologous series (Figure 1 and Figure 2) of isotactic PMAs, including one novel variant, as toxic components of the lipophilic fraction of the Lake Kinneret isolate of *A. ovalisporum*. Although, toxicological evaluation of these compounds was somewhat limited by availability of purified amounts of the toxins—particularly given the relatively low solubility/activity and high exposure concentrations required for the individual congeners—our initial studies suggest both a dose-dependent toxicity relationship (Figure 3) and a relationship between toxicity in the zebrafish embryo model and structural variations (*i.e.*, chain length, degree of methoxylation) of the congeners. Moreover, evaluation of the interactive effects indicates a possible synergism between variants with enhanced developmental toxicity (Figure 4) observed for pair-wise combinations of the PMAs (e.g., **3** and **4**). Toxicological investigation (and required purification of additional material) of the PMAs in the zebrafish model is currently underway and will be presented elsewhere.

To our knowledge, this is the first report of the PMAs as toxic or otherwise bioactive metabolites. It is, accordingly, suggested that these metabolites may not only explain previously observed developmental toxicity [8] of lipophilic fractions from *A. ovalisporum*, but that these previously unrecognized (as toxic) components may contribute to the overall toxicity of this CYN-producing bloom species, and to the cyanobacteria perhaps more generally. Indeed, the prior isolation of these compounds (albeit as non-toxic metabolites) from other cyanobacterial species [13,14,15,16,18] may suggest that these toxic metabolites are relatively widespread, particularly within the cyanobacteria. As such, these findings suggest a need to further investigate these possibly widespread toxic metabolites of the cyanobacteria. To this end, studies to isolate and chemically and toxicologically characterize PMAs from additional cyanobacterial species are currently on-going.
